# Staining of E-selectin ligands on paraffin-embedded sections of tumor tissue

**DOI:** 10.1186/s12885-018-4410-x

**Published:** 2018-05-02

**Authors:** Mylène A. Carrascal, Catarina Talina, Paula Borralho, A. Gonçalo Mineiro, Ana Raquel Henriques, Cláudia Pen, Manuela Martins, Sofia Braga, Robert Sackstein, Paula A. Videira

**Affiliations:** 1 UCIBIO, Departamento Ciências da Vida, Faculdade de Ciências e Tecnologia, Universidade Nova de Lisboa, Lisbon, Portugal; 2 CEDOC, Chronic Diseases Research Center, NOVA Medical School/Faculdade de Ciências Médicas, Universidade Nova de Lisboa, Lisbon, Portugal; 3 Hospital CUF Descobertas, Lisbon, Portugal; 4 Centro Hospitalar de Lisboa Central, EPE e Serviço de Anatomia Patológica, Lisbon, Portugal; 5 Departments of Dermatology and Medicine, Brigham & Women’s Hospital, Harvard Medical School, Boston, USA; 6 Program of Excellence in Glycosciences, Harvard Medical School, Boston, USA; 7 CDG & Allies – Professionals and Patient Associations International Network (CDG & Allies – PPAIN), Caparica, Portugal

**Keywords:** E-selectin ligands, Sialyl-Lewis X, Sialyl-Lewis a, Cancer

## Abstract

**Background:**

The E-selectin ligands expressed by cancer cells mediate adhesion of circulating cancer cells to endothelial cells, as well as within tissue microenvironments important for tumor progression and metastasis. The identification of E-selectin ligands within cancer tissue could yield new biomarkers for patient stratification and aid in identifying novel therapeutic targets. The determinants of selectin ligands consist of sialylated tetrasaccharides, the sialyl Lewis X and A (sLe^X^ and sLe^A^), displayed on protein or lipid scaffolds. Standardized procedures for immunohistochemistry make use of the antibodies against sLe^X^ and/or sLe^A^. However, antibody binding does not define E-selectin binding activity.

**Methods:**

In this study, we developed an immunohistochemical staining technique, using E-selectin-human Ig Fc chimera (E-Ig) to characterize the expression and localization of E-selectin binding sites on paraffin-embedded sections of different cancer tissue.

**Results:**

E-Ig successfully stained cancer cells with high specificity. The E-Ig staining show high reactivity scores in colon and lung adenocarcinoma and moderate reactivity in triple negative breast cancer. Compared with reactivity of antibody against sLe^X/A^, the E-Ig staining presented higher specificity to cancer tissue with better defined borders and less background.

**Conclusions:**

The E-Ig staining technique allows the qualitative and semi-quantitative analysis of E-selectin binding activity on cancer cells. The development of accurate techniques for detection of selectin ligands may contribute to better diagnostic and better understanding of the molecular basis of tumor progression and metastasis.

**Electronic supplementary material:**

The online version of this article (10.1186/s12885-018-4410-x) contains supplementary material, which is available to authorized users.

## Background

Metastasis is initiated when cancer cells leave the primary tumor and disseminate to other parts of the body, where these cells are able to proliferate and form new tumors. The metastasis of vital organs such as the liver, lungs, and bones is commonly initiated from the dissemination of tumor cells through bloodstream. A key and early step of the hematogenous metastasis is the contact of blood-circulating cancer cells with the endothelium. Cancer cells expressing relevant sialofucosylated glycan determinants bind to the endothelial selectins, E- and P-selectin, thereby establishing adhesive interactions with endothelium that resist hemodynamic shear forces. This initial “shear-resistant” adhesion step is requisite for the transendothelial migration of cancer cells from blood into tissues [1]. Since the endothelial selectins are inducible by inflammatory cytokines and expressed constitutively on marrow microvasculature [2, 3], cancer cell binding to selectin is likely to contribute for cancer cell migration to selectin-rich niches, such as inflammation sites and the bone. In addition to their roles in cell adhesion and transendothelial migration, binding to selectins also initiates signal transduction that may promote cancer progression. As an example, in colon cancer, diverse cellular functions such as the activation of SAPK2/p38 [4] and tyrosine phosphorylation of several proteins are induced following engagement of E-selectin ligands [5].

The prototypical selectin binding motif consists of the tetrasaccharide sialyl Lewis X (sLe^X^; NeuAc-α(2,3)-Gal-β(1,4)-[Fuc-α(1,3)]GlcNAc-R), or its stereoisomer sialyl Lewis A (sLe^A^, NeuAc-α(2,3)Gal-β(1,3)-[Fuc-α(1,4)]GlcNAc-R) [5]. The expression of both sLe^X^ and/or sLe^A^ is observed in various cancers in a progressive fashion, increasing in expression from normal tissue to early stage cancer to metastatic disease [6, 7]. In vitro, the expression of sLe^X/A^ by cancer cells correlates with the cancer cell ability to bind endothelial selectins [8]. In tumor tissue, sLe^X/A^ expression has been correlated with the metastasis formation by several cancer types, such as colon carcinoma, lung adenocarcinoma and breast cancer [9–12]. In colorectal cancers, the expression of sLe^X/A^ in the primary lesion is considered a good marker for assessing the metastatic proclivity of colorectal cancer [13]. Indeed, expression of these determinants is also correlated with the extent of malignancy, high incidence of recurrence and with decreased survival of patients [14]. Importantly, the well-recognized clinically-relevant tumor marker CA19–9 is sLe^A^ [15].

Nevertheless, the prognostic value of the detection of the carbohydrates sLe^X^ or sLe^A^, as a sole measure to evaluate selectin ligands, is controversial [16, 17]. The identification of selectin ligands is generally performed using monoclonal antibodies that recognize sLe^X^ and sLe^A^, such as HECA-452, in standardized protocols [9, 10, 18]. Other antibodies developed so far don’t recognize simultaneously both glycans, such as CLEX-1, CA19–9 that recognizes sLe^X^ and CA19–9, respectively. Additionally, it is questionable whether HECA-452 antibody mimic E-selectin binding [19]. Binding to E-selectin itself, is more specific for identification of E-selectin binding activity displayed on specific protein scaffolds [5, 20]. In addition, there are minor sialofucosylated glycans, which are also carbohydrate determinants of E-selectin ligands, that are not recognized by any current monoclonal antibody [21].

In this study, we developed a novel staining protocol for paraffin-embedded slides of colon cancer tissues, using a mouse E-selectin-human Ig Fc chimera (E-Ig), a validated tool to identify E-selectin ligands in human cells [22, 23]. The E-selectin ligand staining protocol described here stains colon adenocarcinoma cells, as well as other cancer tissue, and produces a consistent membrane staining with little background compared to current staining protocols using antibodies against sLe^X^ and sLe^A^.

## Methods

This study used several slides of two cases of colon adenocarcinoma, one case of normal colon tissue, two cases of triple negative breast cancer and two cases of lung adenocarcinoma. Formalin fixed paraffin-embedded tissue are sectioned and placed onto slides using standard paraffin microtomy. The Lab Vision PreTreatment Module (PTM) from Thermo Scientific, is used for de-paraffinization and antigen retrieval on tumor sections. After blocking endogenous peroxidases, slides are stained using a three-step procedure with the E-Ig, anti-CD62E and HRP polymer. All the steps in these protocols take place at room temperature. All reagents used are listed in Table 1. All procedures were performed under the approval of the Ethics Committee of Hospital CUF Descobertas.

1. De-paraffinization and heat-induced antigen retrieval
  1.1. Place 2-μm thick sections of formalin fixed paraffin-embedded tissue on glass slides (Superfrost Plus, Thermo Scientific) made to ensure firm electrostatic attraction of the tissue sections.
  1.2. Dry slides on oven at 37 °C overnight.
  1.3. Prepare the laboratory instrument, Lab Vision PreTreatment Module (PTM) from Thermo Scientific, to perform de-paraffinization and antigen retrieval of tumor tissue:
    1.3.1. Fill each of the PTM tanks with 1.5 L of the Trilogy Pretreatment Solution 1× and program PTM to preheat to 60 °C and heat to 94 °C for 20 min.
    1.3.2. Start the pre-heating cycle and then mount the slides into the racks and place them into the PTM.
    1.3.3. Start the heating cycle, which will heat to 94 °C and then cool down to 60 °C.
    1.3.4. After the heating cycle has finished, take racks out of PTM and wash slides well with distillated water for 1–5 min.
  1.4. Immerse the slides in 70% ethanol for 10 s.
  1.5. Immerse the slides in 96% ethanol for 10 s.
  1.6. Immerse the slides in 100% ethanol for 10 s. Repeat once.
2. Blockade of endogenous peroxidases
  2.1. Place slides in peroxidase block solution (3% H_2_O_2_) for 15 min.
  2.2. Wash the slides with tap water for 1–2 min.
  2.3. Place the slides in a humidity chamber to avoid the drying of the tissue during the entire staining process. Rinse slides with Tris-Buffered Saline solution with 0.1% Tween 20 (TBST), and leave in TBST for 5 min; repeat one more time.
  2.4. Wipe off the excess of solutions around the tissue on the slides. Mark a circle around the tissue with a hydrophobic pen.
  2.5. Rinse slides with TBST and then leave for 5 min in TBST.
3. Staining with chimeric molecules & antibody:
  3.1. Mouse E-selectin-human Ig Fc chimera (E-Ig)
    3.1.1. Incubate slides with 1:300 dilution of recombinant mouse E-selectin-human Ig Fc chimera (E-Ig) in “Diamond: Antibody Diluent”, for 30 min. The final E-Ig concentration is 1.67 μg/mL.
    3.1.2. Rinse slides with TBST, then place in TBST for 5 min. Repeat once.
    3.1.3. Incubate slides with 1:250 dilution of rat anti-mouse E-selectin (CD62E) monoclonal antibody in 100 μL of “Diamond: Antibody Diluent”, for 30 min. The final antibody concentration in TBST is 2 μg/mL.
    3.1.4. Rinse slides with TBST, then place in TBST for 5 min. Repeat once.
    3.1.5. Incubate slides with the “HiDef Detection HRP Polymer System”, which detects the primary antibody by amplification of antibody (Amplifier) followed by HRP polymer (Detector), explicitly:
      3.1.5.1. Incubate slides with “HiDef Detection Amplifier”, for 10 min.
      3.1.5.2. Rinse slides with TBST, then place in TBST for 5 min. Repeat once.
      3.1.5.3. Incubate slides with “HiDef Detection HRP Polymer Detector”, for 10 min.
    3.1.6. Rinse slides with TBST, then place in TBST for 5 min. Repeat once.

**Table 1:** List of commercial reagents used in this study

Name	Company (Country)	Catalog Number	Comments
Lab vision PT Module	Thermo scientific (USA)		
Trilogy Pretreatment Solution	Cell Marque (USA)	920P	Diluted to 1× with ddH_2_O
Ethanol	AGA (Portugal)	4.006.16.00.00	Used in 70% and 96% solution and pure.
Peroxidase block solution	Atom Scientific (United Kingdom)	GPC8054-E	
TBS IHC wash buffer with Tween 20	Cell Marque	935B-09	Diluted to 1× with ddH_2_O
Diamond antibody diluents	Cell Marque	938B-09	
Hi-Def Detection HRP Polymer System	Cell Marque	954D-30	A polymer containing anti-rat Ig Fc conjugated with HRP
DAB Chromogen/Substrate Bulk Pack (High Contrast)	ScyTek Laboratories (USA)	ACV500	A pack of chromogen concentrate, the 3,3′-Diaminobenzidine tetrahydrochloride (DAB) and DAB substrate
Xylene	Klinipath Netherland (The Netherland)	4055.9005	
Mayer’s Hematoxylin	Bio-Optica (Italy)	05–06002/L	
Quick-D Mounting Medium	Klinipath Nederland	7281	
Coverslips	Thermo Scientific	4951PLUS4	
Humidity chamber	Bio-Optica		
E-Ig chimera	R&D Systems (USA)	575-ES-100	Diluted to 1:300 in Diamond: Antibody Diluent
Rat anti-mouse CD62E	BD Biosystems (USA)	550,290	Diluted to 1:250 in Diamond: Antibody Diluent
Anti-sLe^X/A^ (HECA-452)	Biolegend (USA)	321,302	Diluted to 1:50 in Diamond: Antibody Diluent

Notes: All solutions in the staining process should have 2 mM CaCl_2_. As negative control, the same staining protocol run without the E-Ig or without the rat anti-mouse CD62E monoclonal antibody or by adding EDTA (final concentration of 10 mM) to all the solutions (TBST and “Diamond: Antibody Diluent”) during the staining process.

3.2. Staining with anti-sLe^X^ and anti-sLe^A^ antibody HECA-452
  3.2.1. Incubate slides with 1:50 dilution of rat anti-sLe^X^ and -sLe^A^ monoclonal antibody (clone HECA-452) in 100 μL of “Diamond: Antibody Diluent”, for 1 h. The final antibody concentration is 10 μg/mL.
  3.2.2. Rinse slides with TBST, then place them in TBST for 5 min. Repeat once.
  3.2.3. Incubate slides with “HiDef Detection HRP Polymer System”:
    3.2.3.1. Incubate slides with “HiDef Detection Amplifier”, for 10 min.
    3.2.3.2. Rinse slides with TBST, then place in TBST for 5 min. Repeat.
    3.2.3.3. Incubate slides with “HiDef Detection HRP Polymer Detector”, for 10 min.
  3.2.4. Rinse slides with TBST, then place in TBST for 5 min. Repeat once.

4. Chromogenic detection of HRP and Hematoxylin staining
  4.1. Combine 1 part of chromogen concentrate, the DAB, with 20 parts of DAB substrate and mix thoroughly.
  4.2. Apply the mix substrate solution to the slide. Incubate slides for 3 min. Positive staining produces a dark brown reaction product.
  4.3. Immediately after the colour development, wash slides with tap water for 1–2 min.
  4.4. Incubate slides in hematoxylin, which stains nucleic acids, for 3 min.
  4.5. Wash slides with warm tap water for 1–2 min in order to blueing hematoxylin stain.
5. Dehydration and Mounting
  5.1. Immerse the slides in 70% ethanol for 10 s.
  5.2. Immerse the slides in 96% ethanol for 10 s.
  5.3. Immerse the slides in 100% ethanol for 10 s. Repeat once.
  5.4. Immerse the slides in xylene for 10 s. Repeat once.
  5.5. Add one drop of Quick-D mounting medium to the slide and place the coverslip.
6. Tissue slide evaluation
  6.1. Slides were visualised under a light microscope with coupled camera. A semi-quantitative approach was established for tissue slide evaluation, according to Lin and Prichard (2015) recommendations [24]. The scores used in the classification protocol to evaluate cell staining were 0 when negative, 1 if < 25%, 2 if 26–50%, 3 if 51–75% and 4 if > 75% cells were stained. The scores used in the classification protocol to evaluate staining intensity were 0 when no stain was found, 1 if weak, 2 if intermediate, 3 if strong staining intensity was found. The total semi-quantitative value used to quantify E-Ig and antibody staining in this study combined the two above mentioned scores, and was calculated by multiplying each of the two scores.

## Results

**Fig. 1:**
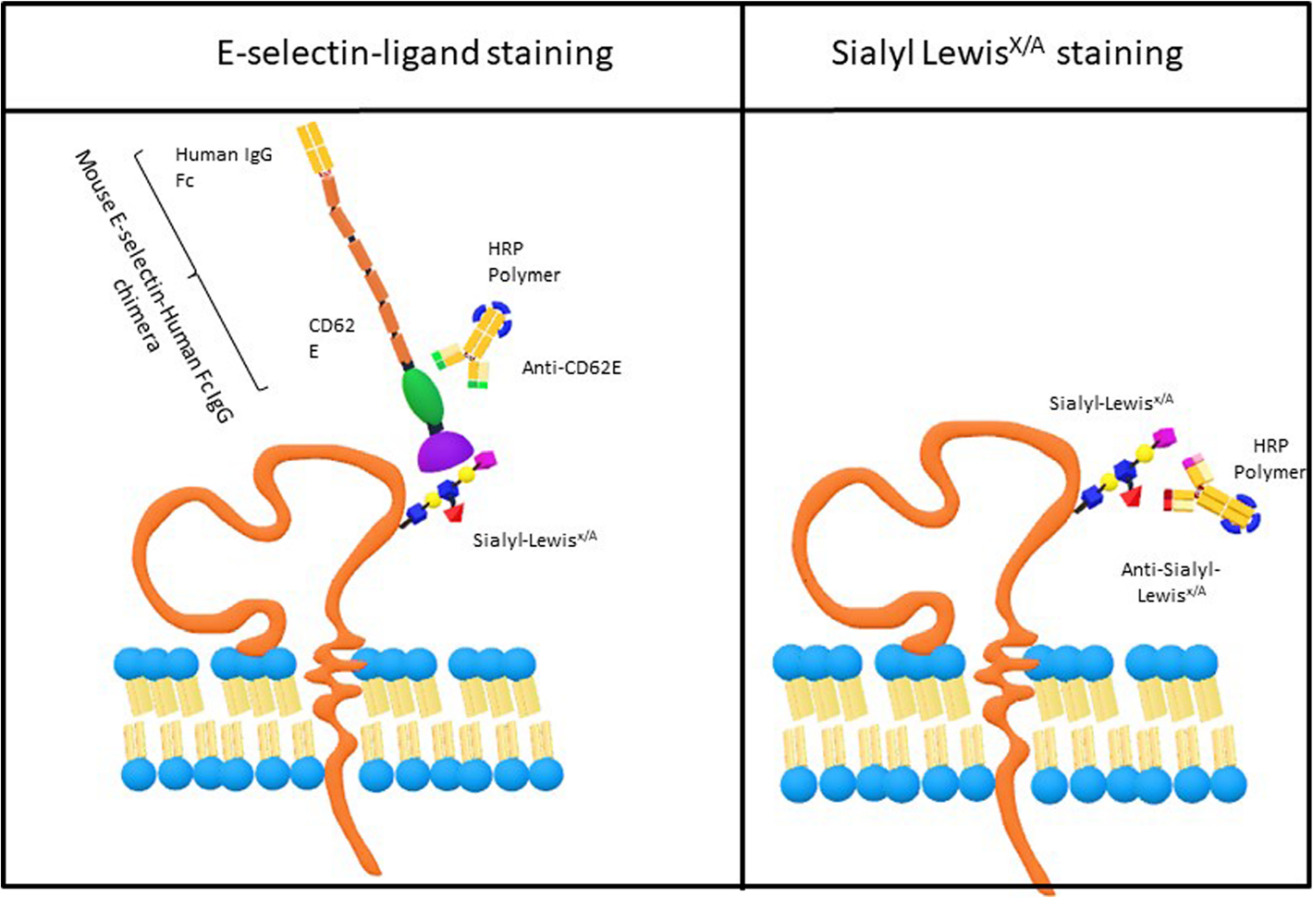
Schematic figure comparing E-Ig and anti-sLe^X/A^ staining technique. E-selectin ligands are recognized by using a three-step staining procedure, where the first staining uses a chimera of mouse E-selectin, i.e. CD62E, with the human IgG Fc (E-Ig). This step is followed by anti-CD62E staining and HRP polymer detection system. The sLe^X/A^ glycan structure is recognized by using anti-sLe^X/A^ antibody followed by HRP polymer detection system

**Fig. 2:**
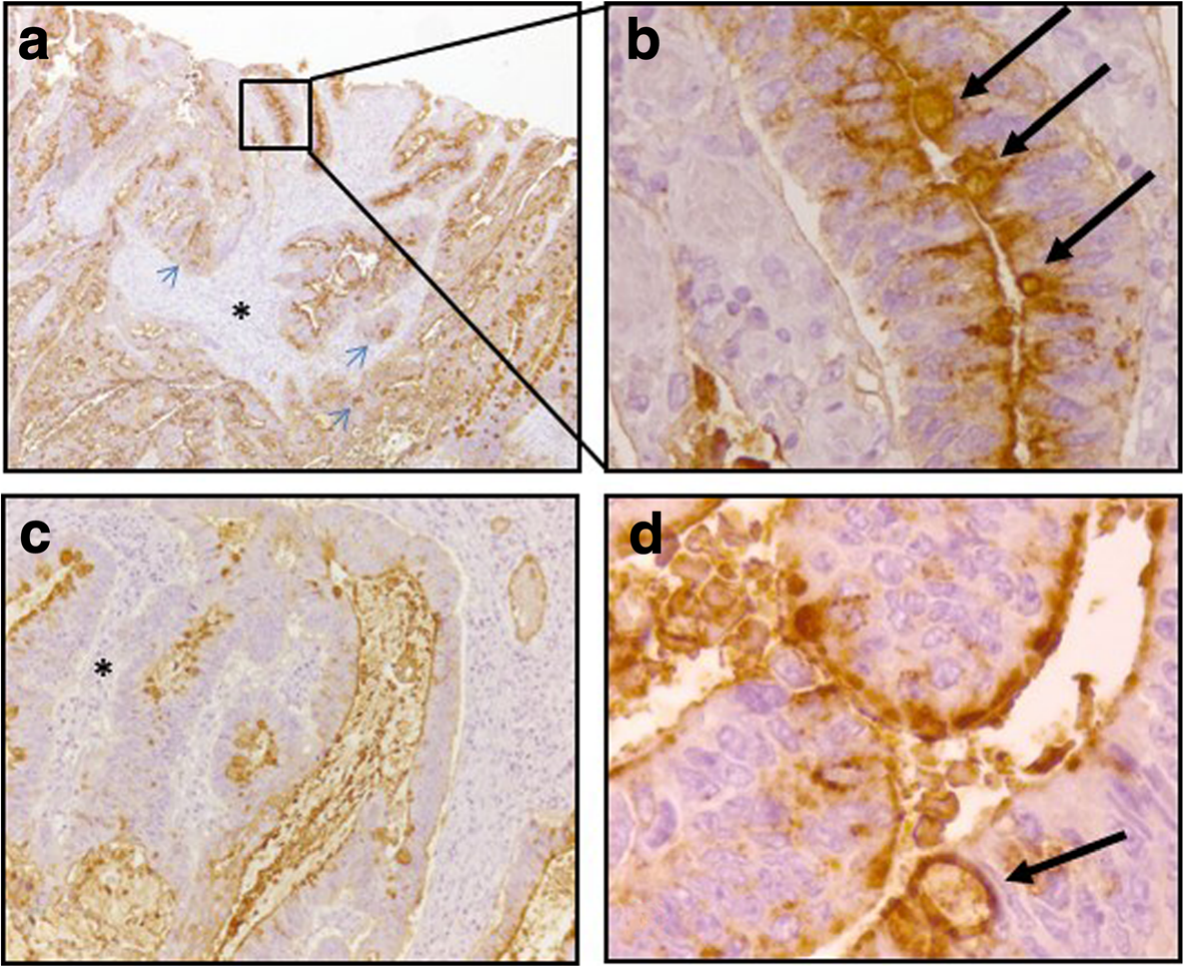
Immunohistochemistry staining of colon adenocarcinoma tissue with E-Ig chimera. Brown color indicates positive reactivity and shows expression of E-selectin ligands in a serial section of the same tissue with 40× magnification (**a**); 400× magnification (**b**), 100× magnification (**c**) and 600× magnification (**d**). **b** is an increased magnification of the boxed area shown in **a**, demonstrating the high reactivity with goblet cells (indicated by black arrows) of the crypts and in particular in their apical pole. In **d** it is highlighted the positive reactivity within the lumens of the crypts and in the cellular cytoplasm (indicated by black arrow). In **a** and **c** the asterisks show the lamina propria with no staining, except for nests of neoplastic cells indicated by the blue arrows

**Fig. 3:**
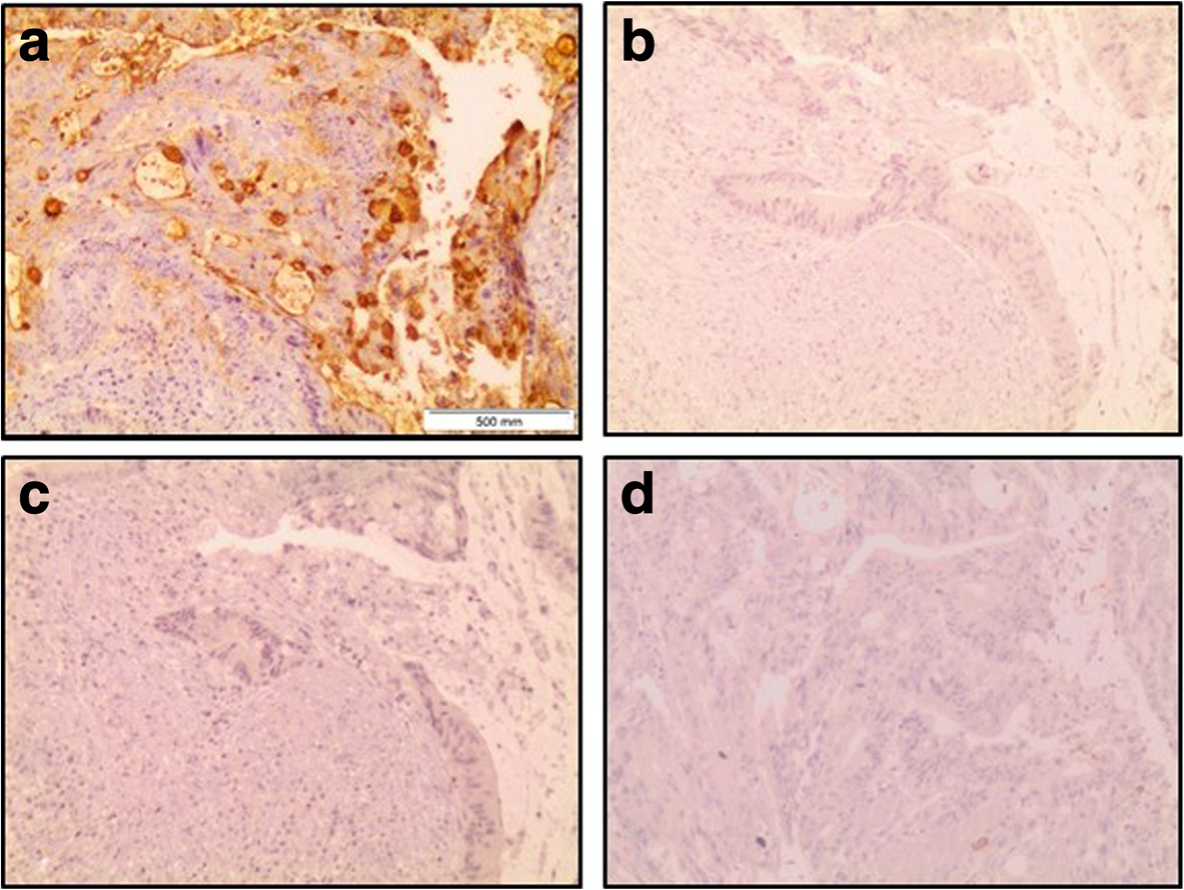
Specificity of the immunohistochemistry staining of colon adenocarcinoma tissue with E-Ig chimera. Images were taken with a 10× magnification, in sequences from the same tissue section of the same paraffin block of tumor tissue. Brown colour indicates positive reactivity and shows expression of E-selectin ligands (**a**). For control staining was performed in the absence of E-Ig (**b**), absence of anti-CD62E monoclonal antibody (**c**) and in presence of a calcium chelant - EDTA (**d**)

In this study, we first tested the use of a chimeric mouse E-selectin fused to the human Fc region of immunoglobulins (i.e., mouse E-selectin-human Ig Fc chimera (E-Ig)) to staining of E-selectin ligands in colon adenocarcinoma tissue. The E-Ig staining technique is a three-step procedure that includes an incubation with E-Ig, followed by incubation against E-selectin (anti-CD62E) and then incubation with a polymer of anti-rat Ig Fc conjugated with horseradish peroxidase (HRP), hereafter named HRP polymer (Fig. 1). As shown in Fig. 2, this technique allowed a successful immunohistological staining demonstrated by the brown staining on cancer tissue, obtained after 3,3′-diaminobenzidine tetrahydrochloride (DAB) color - enzyme detection. The E-Ig staining was strong in colon adenocarcinoma tissue (with a score 3 of cell staining and an intensity of score 4, total score = 12), showing a scattered pattern with stronger reactivity within crypts. The strongest staining signal was on the goblet cells in their apical pole (Fig. 2a-d). The lamina propria showed E-Ig staining exclusively on nests of neoplastic cells (Fig. 2a and c). Staining was not detected (total score = 0) in the control assays, run in the absence of anti-CD62E (Fig. 3a and b), or absence of E-Ig (Fig. 3c), or when assays were performed in presence of a calcium chelator (EDTA) (Fig. 3d), thus confirming the specificity of E-Ig staining.

**Fig. 4:**
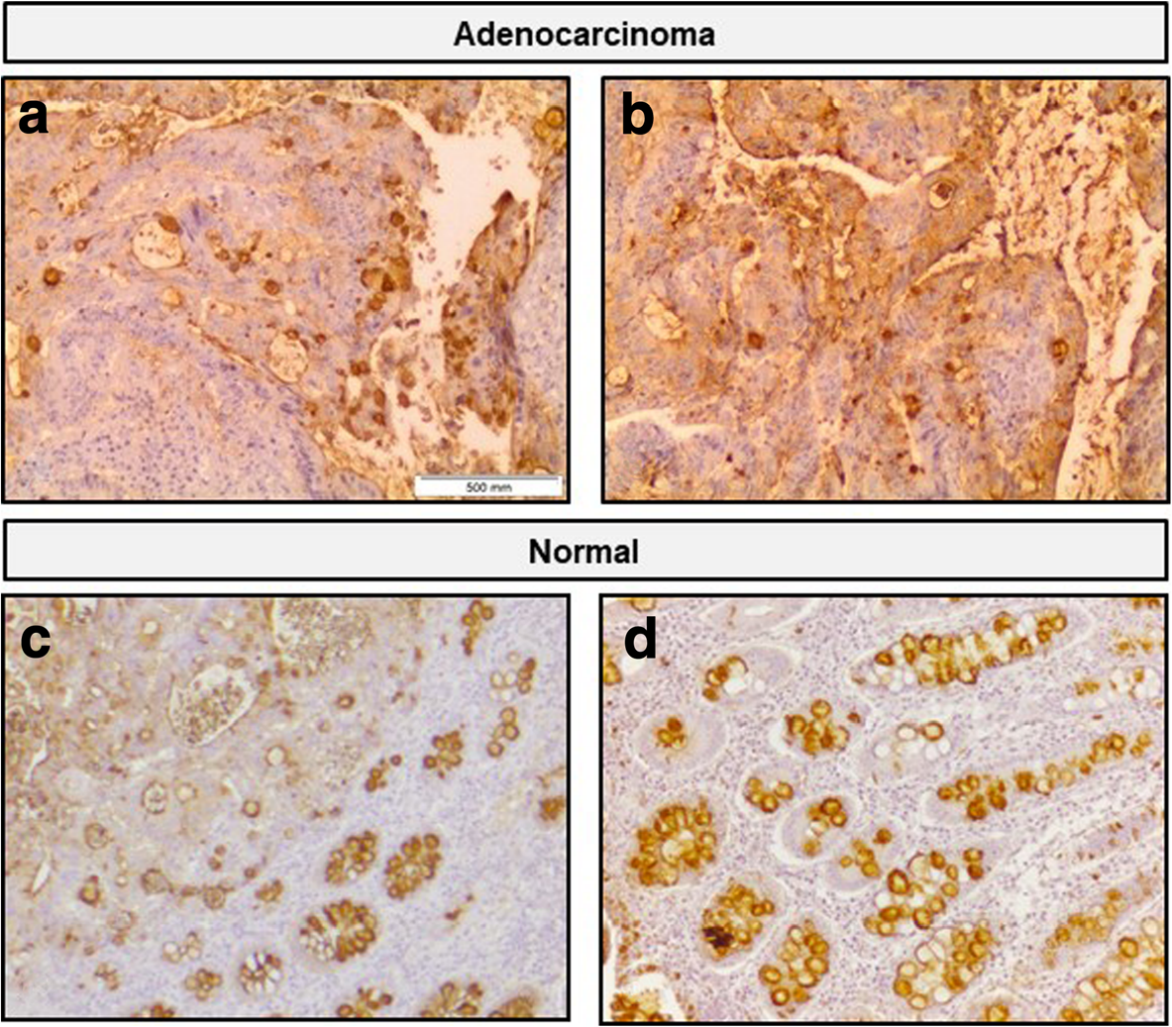
Immunohistochemistry staining of colon adenocarcinoma and normal colon tissue with E-Ig chimera and HECA-452 antibody. The E-Ig chimera that recognizes selectin ligands was used to stain colon adenocarcinoma (**a**) or normal colon (**c**) tissues, with 40× magnification. The HECA-452 antibody, that recognizes sLe^X^ and sLe^A^ glycans, was used to stain colon adenocarcinoma (**b**) or normal colon (**d**) tissues. In case of tumor tissue, images were also taken in sequences from the same tissue section of the same paraffin block of tumor tissue. Brown color indicates E-Ig or HECA-452 reactivity. In adenocarcinoma, the lamina propria showed E-Ig staining exclusively on nests of neoplastic cells, while HECA-452 staining showed positive scattered staining. In normal tissue, both E-Ig chimera and HECA-452 stains the lumens of the crypts and, in particular, the goblet cells

As E-selectin ligands have been inferred by others using antibodies against sLe^X/A^ glycans, we compared the E-Ig staining profile with the immunohistochemical expression profile of colon cancer tissues stained with antibody that recognize both sLe^X^ and sLe^A^, the HECA-452 clone. HECA-452 staining was in general stronger (with a score 3 (or 4) of cell staining and an intensity of score 4, total score = 12 (or 16)). Yet, the major differences were quantitative, as the lamina propria showed scattered HECA-452 staining, which was not exclusive of neoplastic cells nor stained by E-Ig chimera (Fig. 4a, b).

We have also compared staining in normal colon tissue. As shown in Fig. 4, the strongest staining was in goblet cells (Fig. 4c, d) and negligible staining in the lamina propria.

HECA-452 staining was stronger in the goblet cells of the crypt similarly to the histochemical profile with E-Ig staining of normal colon tissue (Fig. 4c, d).

Thus, from the comparison of results obtained from E-Ig or HECA-452 staining protocols, it is possible to conclude that both protocols stain tumor tissue and normal goblet cells. However, there are quantitative differences in tumor staining, as E-Ig chimera staining generates a specific and clearer staining, while HECA-432 staining is scattered, mainly within the lamina propria.

**Fig. 5:**
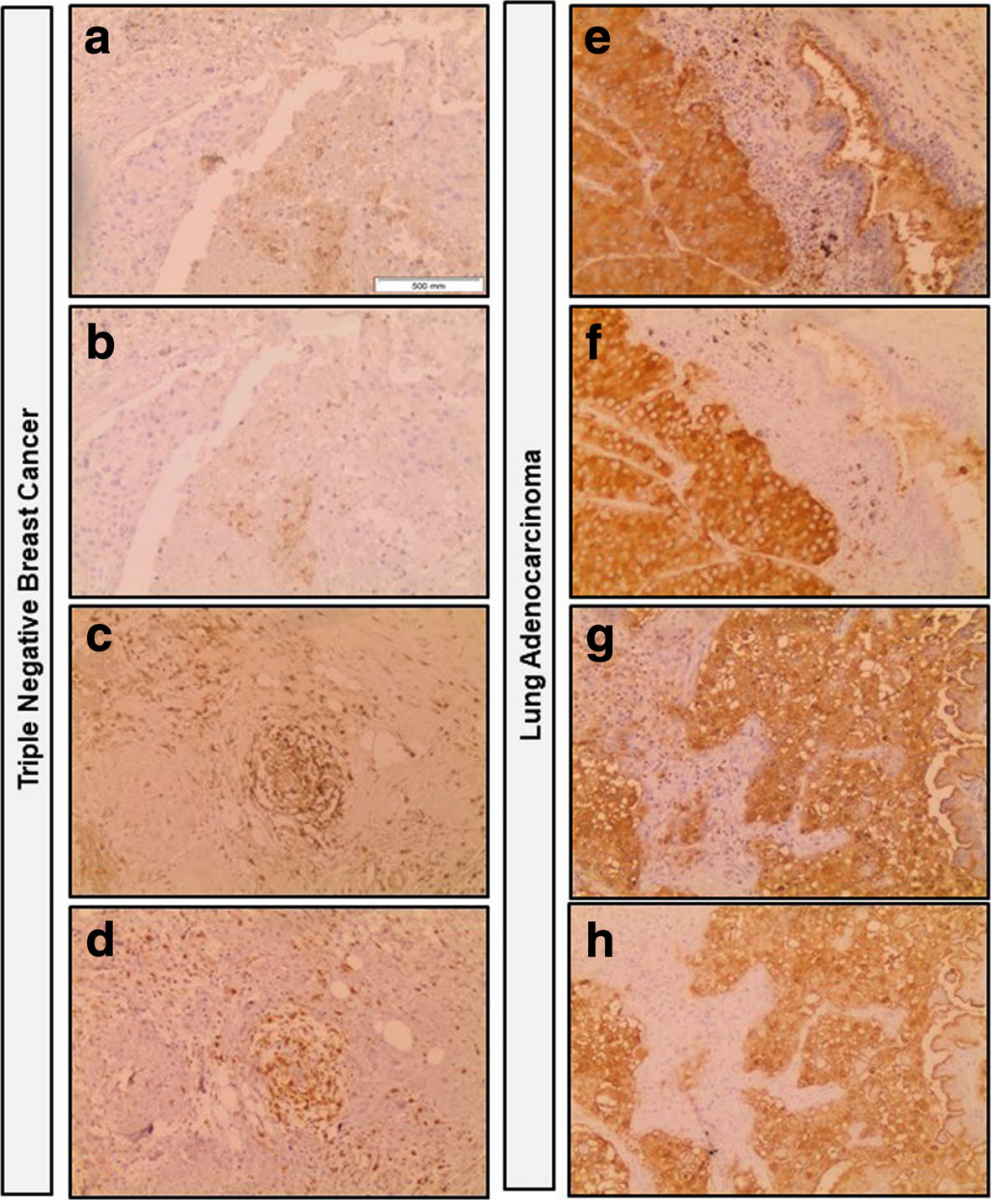
Immunohistochemistry staining of triple negative breast cancer and lung adenocarcinoma tissues with E-Ig chimera and HECA-452 monoclonal antibody. E-Ig was used for staining the E-selectin ligands in triple negative breast cancer (**a** and **c**) and in lung adenocarcinoma (**e** and **g**) tissues. sLe^X^ and sLe^A^ were stained with HECA-452 antibody in triple negative breast cancer (**b** and **d**) and in lung adenocarcinoma (**f** and **h**) tissues. Brown color indicates E-Ig or HECA-452 positive reactivity. Images were taken in sequences from the same tissue section of the same paraffin block of tumor tissue, with a 10× magnification

We have also evaluated the efficacy/universality of E-Ig staining in different types of cancer tissue, namely in triple negative breast cancer and in lung adenocarcinoma. As shown in Fig. 5, E-Ig chimera staining in triple negative breast cancer (Fig. 5a, c) is weaker (with a score 2 of cell staining and an intensity of score 2, total score = 4), in relation to the above-mentioned E-Ig staining in colon adenocarcinoma. Nevertheless, in lung adenocarcinoma E-Ig chimera staining is as strong (Fig. 5e, g) as in colon adenocarcinoma (with a score 3 of cell staining and an intensity of score 4, total score = 12). Staining using E-Ig chimera on either breast or lung adenocarcinoma were slightly stronger with more clear staining of neoplastic tissue and borders, than respective HECA-452 staining (Fig. 5b, d, f, h).

## Discussion

Immunohistochemical staining of selectin ligands has been inferred in prior studies for a variety of cancers, based on the expression of sLe^X/A^, detected by appropriate antibodies [9, 10, 18]. These studies have highlighted that the aberrant expression of sLe^X/A^ epitopes by cancer cells is usually associated with higher propensity for cancer progression and metastization. For instance, in gastric cancer, sLe^X^ expression is an independent risk factor for liver metastasis [9]. In mammary carcinoma, sLe^X^ expression is associated with a higher risk of metastasis [10], and in prostatic carcinoma, sLe^X^ is also associated with poor prognosis [18]. However, despite the fact that expression of sLe^X/A^ is closely associated with selectin ligand binding, it is not in itself predictive of E-selectin ligand activity [19].

Here, we have provided evidence of successful staining of paraffin-embedded tissue with a three-step procedure using an E-selectin chimera (E-Ig). The three-step procedure staining using E-Ig is able to amplify the signal intensity to levels comparable to those obtained using antibodies for immunohistochemistry. Moreover, the use of this E-Ig staining strategy E-Ig staining was more specific for cancer cells, compared with anti-sLe^X/A^ antibody staining that presented a more diffuse pattern especially with non-specific staining in the lamina propria. These relative differences may be due to the fact that while sLe^X/A^ serves as a binding determinant, only the clustered display of these tetrasaccharides on specific protein and/or lipid scaffolds determines E-selectin ligand activity [25]. The scaffold is necessary to create sufficient sLe^X^ and/or sLe^A^ density to engender E-selectin binding [5, 20, 26]. Notably all available anti-sLe^X/A^ antibodies, such as HECA-452, cannot block E-selectin binding [27] and blockade of E-selectin, with anti-E-selectin antibodies, is much more effective in blocking cell adhesion to endothelium [28, 29].

The E-Ig staining recated to goblet cells, in both normal and tumor tissue, and in particular to their apical membrane. This is consistent with the fact that goblet cells are the main producers of mucus in colon, consisting essentially of mucins, which are glycosylated and are well known E-selectin ligands in colon cancer [30, 31]. Nevertheless, the E-selectin staining method here described retrieves a more specific staining with less background signal around the goblet cells. This improvement allows better evaluation of the tissue structure, as well as the relative analysis of the quantity of granules that are inside it, which may have clinical and diagnostic significance.

The detection of neoplastic cells based on E-Ig chimera staining was also effective in other types of cancer, besides colon adenocarcinoma, namely in triple negative breast cancer and in lung adenocarcinoma, which allows us to conclude that it is a universal technique. Interestingly, the staining levels of E-selectin ligands are consistent with the degree of mucus production of these tissues. In fact, as in the gastrointestinal epithelium, the respiratory epithelium also has a significant number of goblet cells that are related to mucins production.

The immunohistochemical staining with E-Ig here described was performed using the Lab Vision PTM, which combines deparaffinization, rehydration, and unmasking in one step. As an alternative, classic methods of de-paraffinization can be used, and citrate buffer can also be utilized during 30 min at 98 °C to perform antigen retrieval. Blockade of non-specific binding is critical to obtain specific results. In this protocol, Tween 20 containing buffers, such as TBST has been used and all antibodies/chimera dilutions were performed using Diamond antibody diluent, which contains bovine serum albumin, as blocking agent. As an alternative, antibody and E-Ig dilution can be performed in TBST following a pre-blocking step with 5% BSA in TBST for 10 min, thus being necessary to adjust the dilution of E-Ig and rat anti-mouse CD62E antibody as appropriate. One of the critical aspects of the E-Ig staining protocol described here is incubation in the presence of calcium, since the binding of E-selectin receptor to E-selectin ligands is calcium-dependent. Therefore, it’s necessary to add up to 2 mM of CaCl_2_ to both staining and washing buffer solutions. Binding specificity must be always confirmed using EDTA chelation, which has the ability to “sequester” calcium, diminishing its reactivity and inhibiting the binding of E-Ig to E-selectin ligands. In this protocol, we verified that adding 10 mM EDTA to both staining and washing buffer solutions, there was an efficient inhibition of the E-Ig staining to colon tissues. During E-Ig staining, the use of a secondary antibody, rat anti-mouse CD62E, is critical since the HiDef amplification and HRP polymer solutions bind to rat Ig Fc regions and do not recognize the human Ig Fc region contained in E-Ig chimera molecules. Importantly, non-specific binding of the secondary antibody must always be excluded. As an alternative to HiDef amplification and HRP polymer solutions, one can use an anti-rat IgG antibody conjugated with HRP.

In addition, the protocol can be further adapted to other techniques such as immunofluorescence. This would depend on the reporter system used, i.e., the reporter conjugated into the final step. In Additional file 1: Figure S1, it is possible to observe the identification of E-selectin ligands in tumor CF1T_cells [23], by adapting the present protocol to include a secondary antibody conjugated to a fluorescent reporter.

In our view, the use of E-Ig chimera staining is more effective than sLe^X/A^ antibodies since the staining is done directly on the ligands which effectively have the potential to bind to E-selectin, including sialofucosylated ligands which are not detected by the generally used antibodies, like HECA-452. Although the assays conducted by immunohistochemistry are limited as they do not directly address the capacity of cells to bind to endothelium, when they are circulating in the vascular flow, the correct identification of E-selectin ligands with E-Ig chimera in the tissue, can simulate the potential of these cells to metastazise. In this way, the analysis of the metastization potential of the tissue is done in a more integrated way and considering the physiological aspects of ligand binding to E-selectin.

## Conclusions

The E-Ig staining technique here described allowed for qualitative and semi-quantitative analysis of E-selectin ligands expression and location on colon adenocarcinoma cells and it may be applied to stain other tissues. The development of cancer-specific immunohistochemical staining methods is of paramount relevance, since the tumor tissue samples are often obligatory stored and used in the form of paraffin-embedded blocks, for diagnosis and for patient stratification. As E-selectin ligands play a fundamental role in the metastatic processes of cancer cells of several cancer types, including various adenocarcinomas, this staining technique will also facilitate our understanding of the molecular basis of tumor progression and metastasis.

## Additional file

Additional file 1: **Figure S1.** Example of application of staining of E-selectin ligands by Immunofluorescence. Staining of E-selectin ligands in CF1_T cells. The breast cancer cell line CF1_T has a high content of E-selectin ligands and it was obtained and cultured as described by Carrascal et al. (2017). Cells were cultured on glass coverslips overnight and then fixed with 3.7% paraformaldehyde. After blocking with 1% bovine serum albumin, cells were stained with E-Ig chimera in the presence of 2 mM CaCl_2._ The final step included anti-human Ig antibody conjugated with fluorescein (FITC, green), in the presence of PBS containing 2 mM CaCl_2_ (A). Control experiments were processed in the absence of CaCl_2_ (B). After permeabilization with 0.1% TritonX-100, F-actin was stained with Alexa Fluor 568 phalloidin (Molecular Probes, Leiden, Netherlands). Images were acquired with a Leica TCS SP2 AOBS confocal microscope. A representative cross-section confocal images were selected after Z-stacking. (PPTX 230 kb)

## Abbreviations

E-Ig: E-selectin-human Ig Fc chimera
sLe^A^: sialyl Lewis A;
sLe^X^: sialyl Lewis X;
