# Supplementary material for: Staining of E-selectin ligands on paraffin-embedded sections of tumor tissue
Source: BMC Cancer. 2018 May 2;18:495. doi: 10.1186/s12885-018-4410-x (PMC5930952; doi:10.1186/s12885-018-4410-x)
Supplement: Supplementary file 1 — Figure S1. Example of application of staining of E-selectin ligands by Immunofluorescence. Staining of E-selectin ligands in CF1_T cells. The breast cancer cell line CF1_T has a high content of E-selectin ligands and it was obtained and cultured as described by Carrascal et al. (2017). Cells were cultured on glass coverslips overnight and then fixed with 3.7% paraformaldehyde. After blocking with 1% bovine serum albumin, cells were stained with E-Ig chimera in the presence of 2 mM CaCl2. The final step included anti-human Ig antibody conjugated with fluorescein (FITC, green), in the presence of PBS containing 2 mM CaCl2 (A). Control experiments were processed in the absence of CaCl2 (B). After permeabilization with 0.1% TritonX-100, F-actin was stained with Alexa Fluor 568 phalloidin (Molecular Probes, Leiden, Netherlands). Images were acquired with a Leica TCS SP2 AOBS confocal microscope. A representative cross-section confocal images were selected after Z-stacking. (PPTX 230 kb) [file 12885_2018_4410_MOESM1_ESM.pptx]

## Slide 1
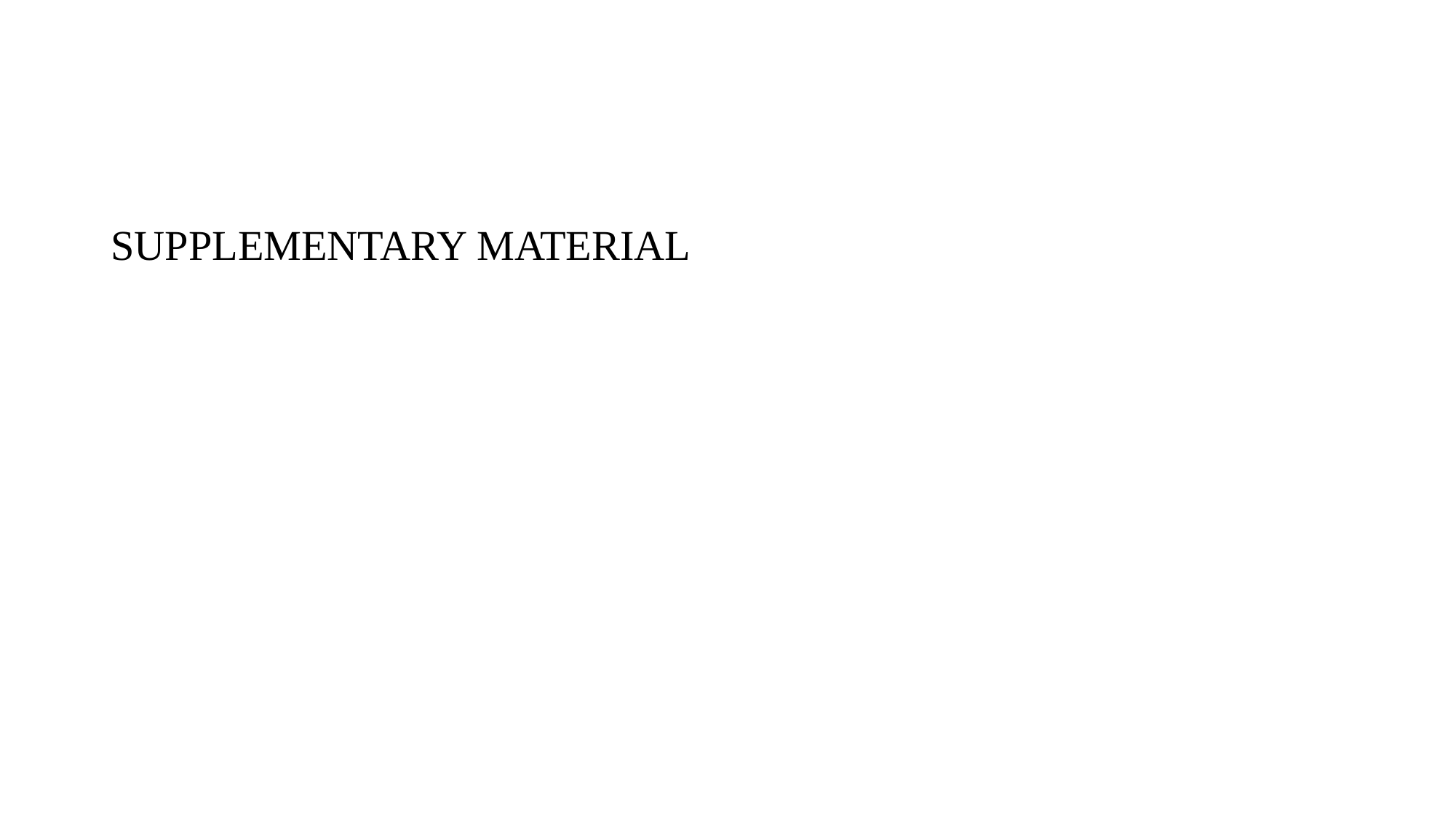

SUPPLEMENTARY MATERIAL

## Slide 2
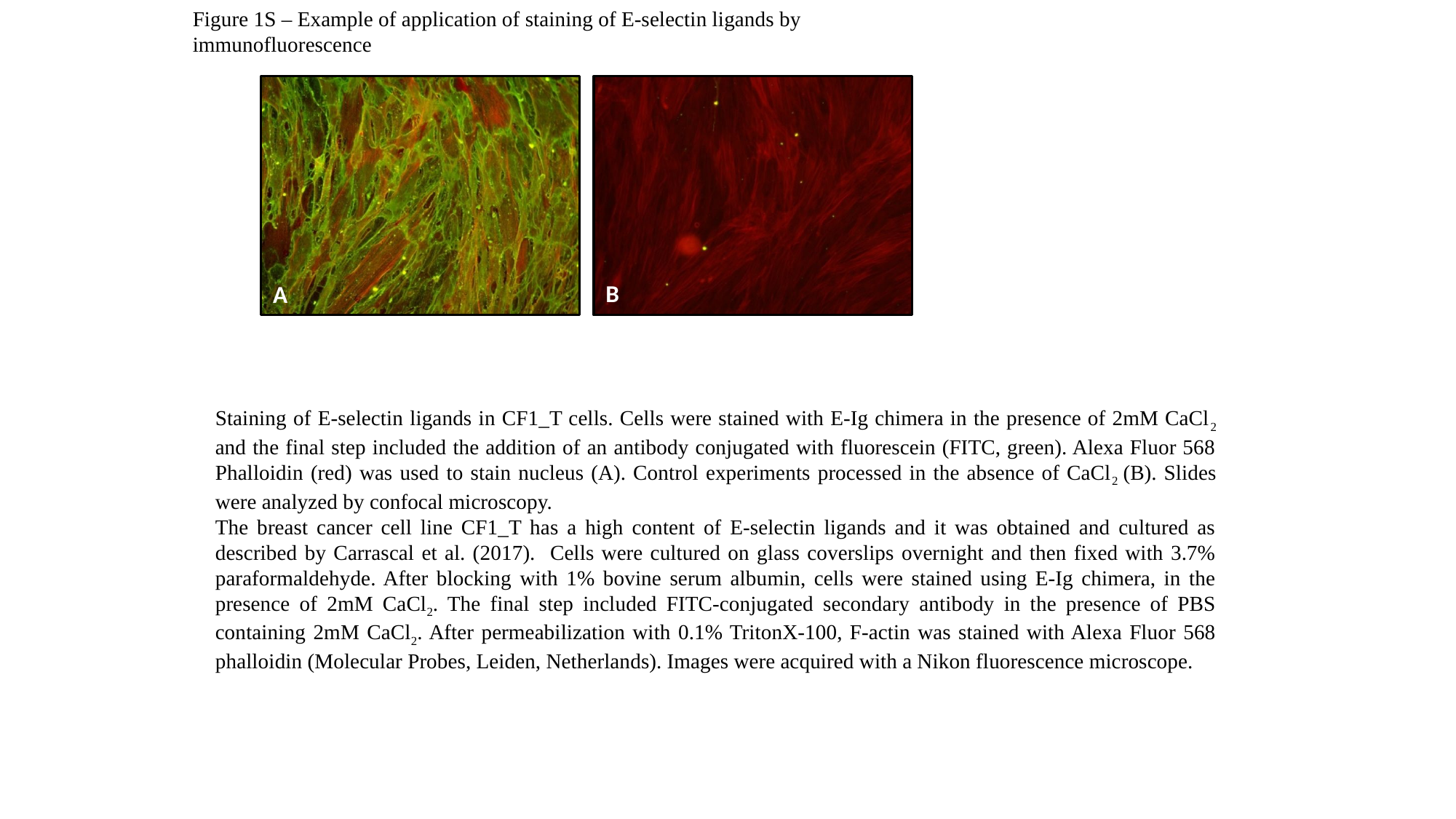

Figure 1S – Example of application of staining of E-selectin ligands by immunofluorescence
B
A
Staining of E-selectin ligands in CF1_T cells. Cells were stained with E-Ig chimera in the presence of 2mM CaCl2 and the final step included the addition of an antibody conjugated with fluorescein (FITC, green). Alexa Fluor 568 Phalloidin (red) was used to stain nucleus (A). Control experiments processed in the absence of CaCl2 (B). Slides were analyzed by confocal microscopy.
The breast cancer cell line CF1_T has a high content of E-selectin ligands and it was obtained and cultured as described by Carrascal et al. (2017). Cells were cultured on glass coverslips overnight and then fixed with 3.7% paraformaldehyde. After blocking with 1% bovine serum albumin, cells were stained using E-Ig chimera, in the presence of 2mM CaCl2. The final step included FITC-conjugated secondary antibody in the presence of PBS containing 2mM CaCl2. After permeabilization with 0.1% TritonX-100, F-actin was stained with Alexa Fluor 568 phalloidin (Molecular Probes, Leiden, Netherlands). Images were acquired with a Nikon fluorescence microscope.
